# Zopfiellamides C and D, New Decalin-Type Tetramic Acid Derivatives from the Marine-Derived Fungus *Aspergillus* sp. NF666

**DOI:** 10.3390/molecules30071502

**Published:** 2025-03-28

**Authors:** Fangwen Jiao, Tianyu Liu, Kaiwei Wang, Shuai Li, Ruihua Jiao, Wei Lin

**Affiliations:** 1 Department of Pathogen Biology, School of Medicine, Nanjing University of Chinese Medicine, Nanjing 210023, China; 17861203185@163.com (T.L.); zhuxuansiyu@njucm.edu.cn (K.W.); 19895576395@163.com (S.L.); 2 Jiangsu Key Laboratory of Medicinal Substance and Utilization of Fresh Chinese Medicine, Nanjing 210023, China; 3 State Key Laboratory of Dao-di Herbs, Beijing 100700, China; 4 State Key Laboratory of Pharmaceutical Biotechnology, Institute of Functional Biomolecules, School of Life Sciences, Nanjing University, Nanjing 210023, China; rhjiao@nju.edu.cn

**Keywords:** tetramic acid, zopfiellamides, marine fungus, *Aspergillus* sp., structure elucidation, antibacterial activity, biosynthesis

## Abstract

Two new decalin-tetramic acid hybrid metabolites, zopfiellamides C (**1**) and D (**2**) were isolated from the marine-derived fungus *Aspergillus* sp. NF666. The structure determination was accomplished on the basis of HRESIMS and NMR spectral data analyses including COSY, HSQC, HMBC, and NOESY experiments. Both isolated metabolites (**1** and **2**) exhibited significant growth inhibition against four clinically relevant bacterial strains with minimum inhibitory concentration (MIC) values of about 12.5 μΜ. Moreover, we proposed a plausible biosynthetic pathway of zopfiellamide D (**2**) in this work.

## 1. Introduction

Natural products derived from the secondary metabolism of microorganisms (bacteria and fungi) represent a large family of chemical molecules, and their structural diversity and attractive biological activities make them interesting as a source for the identification of novel antibiotics and other drug leads [1,2,3]. Marine-derived fungi have been considered as an abundant and important microbial resource for producing novel natural products [4,5]. As an essential part of marine microorganisms, *Aspergillus* is a diverse genus of filamentous fungi with approximately 446 known species. Species of this genus are known to be a rich source of secondary metabolites, including polyketides, alkaloids, terpenoids, peptides, sterols, fatty acids, and other compounds, displaying a variety of pharmacological activities such as antimicrobial, cytotoxic, anti-inflammatory, and antioxidant activities [6,7,8]. A typical example is lovastatin, produced by *Aspergillus terreus*, which is a well-known cholesterol-lowering drug on the market [9]. Even after much attention and investigations spanning over two decades, this genus still holds great potential to provide metabolites with new structures and remarkable biological activities.

**Figure 1:**
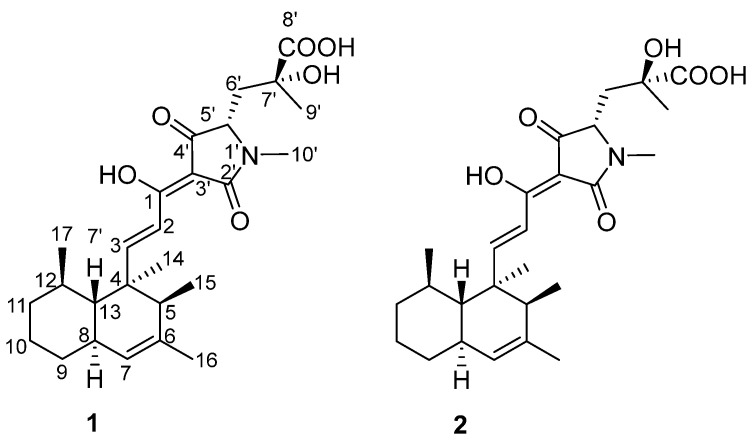
Structures of the isolated compounds **1** and **2**.

Natural products that contain the decalin-tetramic acid motif have been studied extensively, and many of them possess excellent biological activities, for example, the HIV-1 integrase inhibitors equisetin and phomasetin [10,11,12]. Partial decalin-tetramic acid derivatives featuring an unnatural 4,4-disubstituted glutamic acid unit, such as Sch 210971 [13,14], Sch 210972 [15], and JBIR-22 [16,17], usually act as protein–protein interaction inhibitors that are of relevance to proteasome assembly. The complex chemical structures and stereochemistry of decalin-type tetramic acid compounds have prompted extensive synthetic and biosynthetic studies. Marine-derived *Aspergillus* sp. NF666, which was isolated from marine mud in the South China Sea, was found to produce novel azaspirenes A–E and siderophores in our previous study [18]. In our ongoing investigations, two new peaks with characteristic UV absorption bands around 280 nm were detected by HPLC analysis of the EtOAc extract. Subsequent chemical investigation led to the identification of two novel antibacterial decalin-type tetramic acid metabolites, zopfiellamides C (**1**) and D (**2**) (Figure 1). Details of the isolation, structural elucidation, antibacterial activity, and proposed biosynthetic pathway of zopfiellamides C (**1**) and D (**2**) are reported herein.

## 2. Results

### 2.1. Structural Elucidation of the Compounds

Compound **1** was isolated as a colorless, amorphous powder, and the molecular formula was determined to be C_26_H_37_NO_6_ on the basis of the HRESIMS ion peak at *m/z* [M + H]^+^ 460.2708 (calcd. for C_26_H_38_NO_6_, 460.2699), indicating nine degrees of unsaturation. The direct connectivity between each proton and carbon was established by the HSQC spectrum; the ^13^C and ^1^H NMR data for **1** are shown in Table 1. The NMR spectral data of **1** revealed characteristic signals of four methylenes [*δ*_H_ 1.17 (1H, m, H-9), 1.79 (1H, m, H-9), 1.73 (2H, m, H-10), 1.22 (1H, m, H-11), 1.68 (1H, m, H-11), 2.02 (1H, m, H-6′), and 2.39 (1H, dd, *J* = 2.3, 14.7 Hz, H-6′); *δ*_C_ 34.8 (C-9), 27.6 (C-10), 39.0 (C-11), and 39.2 (C-6′)], six methyls [*δ*_H_ 1.18 (3H, s, H-14), 1.01 (3H, d, *J* = 7.0 Hz, H-15), 1.67 (3H, s, H-16), 0.82 (3H, d, *J* = 5.7 Hz, H-17), 1.47 (3H, s, H-9′), and 2.98 (3H, s, H-10′); *δ*_C_ 17.9 (C-14), 16.8 (C-15), 22.3 (C-16), 23.1 (C-17), 27.2 (C-9′), and 27.1 (C-10′)], five methines [*δ*_H_ 1.56 (1H, q, *J* = 6.8 Hz, H-5), 1.83 (1H, m, H-8), 1.44 (1H, m, H-12), 1.44 (1H, m, H-13), and 3.93 (1H, d, *J* = 6.7 Hz, H-5′); *δ*_C_ 50.9 (C-5), 41.4 (C-8), 36.5 (C-12), 49.3 (C-13), and 65.0 (C-5′)], as well as two trans olefinic methine protons [*δ*_H_ 7.11 (d, *J* = 16.1 Hz, H-2), 7.62 (1H, d, *J* = 16.1 Hz, H-3); *δ*_C_ 115.7 (C-2), and 164.9 (C-3)] and a single olefinic methine proton [*δ*_H_ 5.13 (1H, s, H-7); *δ*_C_ 126.0 (C-7)].

**Figure 2:**
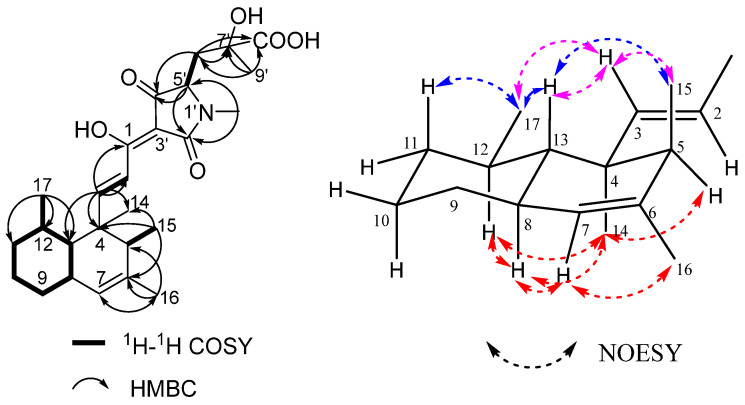
The key ^1^H-^1^H COSY, HMBC (**left**), and NOESY (H→H, (**right**)) correlations of compound **1**.

The continuous correlations in ^1^H-^1^H couplings revealed the presence of the 4, 5, 6, 12-tetramethyldecalin moiety. In the HMBC spectrum of **1**, the correlations from olefinic methine protons H-3 (*δ*_H_ 7.62) to C-4 (*δ*_C_ 43.6) and H-2 (*δ*_H_ 7.11) to C-1 (*δ*_C_ 174.8) suggested that the *α*, *β*-unsaturated carbonyl moiety was substituted on the decalin moiety. An N-methyl proton H-10′ (*δ*_H_ 2.98) displayed HMBC correlations to an amide carbonyl carbon C-2′ (*δ*_C_ 174.2) and a methine carbon C-5′ (*δ*_C_ 65.0), which was ^1^H-^1^H spin-coupled to methylene protons H-6′ (*δ*_H_ 2.02, 2.39). The methyl protons H-9′ (*δ*_H_ 1.47) as well as methylene protons H-6′ respectively had HMBC correlations to a quaternary carbon C-7′ (*δ*_C_ 74.0) and a carboxylic carbonyl carbon C-8′ (*δ*_C_ 177.3), showing that the carboxylic acid residue was located at C-7′. The HMBC correlations from H-5′ and H-6′ to C-4′ (*δ*_C_ 197.4) revealed that C-4′ was connected to C-5′. By taking into consideration a remaining quaternary olefinic carbon C-3′ (*δ*_C_ 100.1) together with the typical ^13^C NMR chemical shifts of C-4′ and the *α*, *β*-unsaturated carbonyl carbon C-1, the unit from N-1′ to C-5′ forms a pyrrolidinone moiety (Figure 2). According to the molecular formula of **1**, we established the planar structure of **1** as shown in Figure 1. Compound **1** has a very similar structure to the other pyrrolidinone derivatives zopfiellamides A and B [19], so this compound was named as zopfiellamide C.

The relative configuration of the decalin part was found to be the same as that of zopfiellamide A as deduced from the NOESY spectra. The NOESY correlations observed between H-8 (*δ*_H_ 1.83, m)/H-12 (*δ*_H_ 1.44, m), H-8/H-7 (*δ*_H_ 5.13, s), H-8/Me-14 (*δ*_H_ 1.18, s), H-7/Me-16 (*δ*_H_ 1.67, s), H-5 (*δ*_H_ 1.56, q)/Me-14, and Me-14/H-12 revealed that these protons were directed toward the same face (*α*); while H-13 (*δ*_H_ 1.44, m) correlated with Me-15 (*δ*_H_ 1.01, d) and Me-17 (*δ*_H_ 0.82, d), Me-17 correlated with H-11ax (*δ*_H_ 1.22, m), and thus they were *β*-oriented. In addition, the strong NOESY correlations from trans olefinic H-3 (*δ*_H_ 7.62, d) to H-13, Me-17, and Me-15 showed that they are close to each other in spatial position (Figure 2).

**Figure 3:**
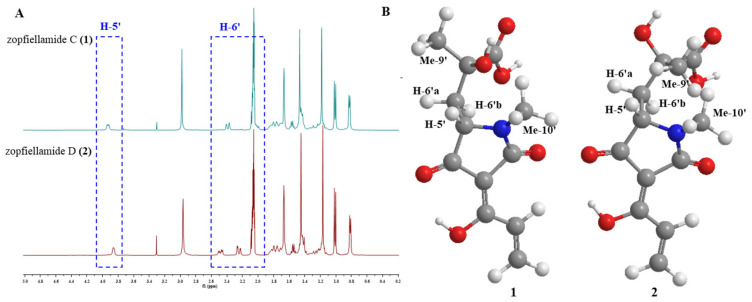
(**A**) The chemical shift discrepancy of CH-5′ and CH_2_-6′ in ^1^H NMR spectrum in zopfiellamides C (**1**) and D (**2**); (**B**) the chemical simulation of tetramic acid moiety in zopfiellamides C (**1**) and D (**2**) with Chem 3D.

The molecular formula of compound **2** was determined to be C_26_H_37_NO_6_ according to the [M + H]^+^ ion at *m/z* 460.2699 (calcd. for C_26_H_38_NO_6_, 460.2699) in its HRESIMS, which had the same molecular formula as **1**. The similar ^1^H and ^13^C NMR spectra provided further supportive evidence for the structure of **2**, and the only noticeable differences were found in the side chain of the tetramic acid part. The significant differences of chemical shifts at C-7′, C-9′, and C-10′ (+0.8 ppm) in comparison to **1** indicated a change of the side chain attached to the tetramic acid ring in **2**. Scrutiny of the NOESY data for the tetramic acid part revealed that H-5′ has stronger NOESY correlation with Me-10′ than Me-9′ in both compounds, indicating that the relative configurations of C-5′ are both *S*. In addition, one H-6′ [2.48 (dd, *J* = 6.0, 15.0 Hz)] was shifted downfield (+0.46 ppm), with the other [*δ*_H_ 2.25 (dd, *J* = 2.9, 15.0 Hz)] shifting upfield (−0.14 ppm). Meanwhile, H-5′ (*δ*_H_ 3.86) was also shifted upfield (−0.07 ppm) in **2** (Figure 3A). The observation of a large chemical shift difference at H-6′ and H-5′ due to the steric effects of hydroxyl and carboxylic acid strongly suggested the opposite stereochemistry arrangements of hydroxyl and carboxylic acid groups at C-7′ in **2**. Combined with chemical simulation (Figure 3B), only when the configuration of C-7′ changes from *R* to *S* will the above changes in chemical shift occur. The discrepancy in NMR spectra could only be explained by a different configuration at C-7′, so they should be 7′-epimers of each other. Hence, compound **1** was determined to have 5′*S*, 7′*R* stereochemistry, while compound **2** has 5′*S*, 7′*S* stereochemistry, as shown in Figure 1.

### 2.2. Antibacterial Activities

The isolated metabolites were evaluated for their antibacterial activities. Compounds **1** and **2** exhibit good antibacterial activities against a panel of bacterial pathogens including *Pseudomonas aeruginosa*, *Staphylococcus aureus*, *Escherichia coli*, and *Pectobacterium carotovorum* subsp. *Carotovorum* with MIC values of about 12.5 μΜ (Table 2). It seems that the configuration at C-5′ has strong correlation with the antibacterial activities, as the epimer of **1** (i.e., **2**) was less active than **1**.

### 2.3. Proposed Biosynthesis Pathway

**Figure 4:**
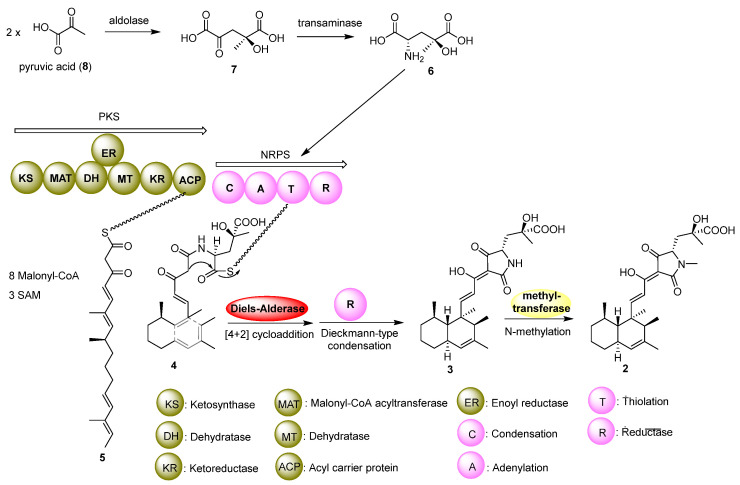
Proposed biosynthetic pathway of zopfiellamide D (**2**).

At last, we proposed the biosynthetic pathway of zopfiellamide D (**2**). As proposed, the core decalin-tetramic acid structure is synthesized by dual-modular polyketide synthase–nonribosomal peptide synthetase (PKS-NRPS) proteins [20]. In this large hybrid enzyme, the PKS utilizes malonyl-CoA and *S*-adenosyl-L-methionine (SAM) to synthesize the linear polyketide intermediate (**5**) with the assistance of stand-alone enoyl reductase (ER), which could specifically reduce a nascent polyketide backbone double bond [21]. Then, the NRPS adds an unusual amino acid, *γ*-hydroxymethyl-L-glutamic acid (**6**), which is derived from two molecules of pyruvic acid (**8**) to form intermediate **7**; different configurations of **7** may be formed by an aldolase. Then, a Diels–Alderase would bind to and lock the straight-chain polyketide intermediate **4** in a specific conformation to promote the [4+2] cycloaddition reaction and control the stereoselectivity of the reaction [22,23]. The terminal reductase domain (R) is proposed to catalyze the tetramate moiety-forming via Dieckmann-type condensation and release the product [24]. Finally, N-methylation could yield zopfiellamides C (**1**) and D (**2**) (Figure 4).

## 3. Materials and Methods

### 3.1. General Experiment Procedure

All analytical and semi-preparative HPLC processes were carried out on an Agilent 1220 HPLC system with a DAD detector equipped with a Poroshell 120 EC-C18 column (Agilent Technologies, Waldbronn, Germany) and a Zorbax Eclipse XDB column (C-18, 9.4 × 250 mm, 5 μm, Agilent Technologies, Waldbronn, Germany), respectively. NMR spectra were obtained on a Bruker Avance III 400 spectrometer at 400 MHz for ^1^H and 100 MHz for ^13^C nuclei (Bruker, Zurich, Switzerland). HRESIMS data were measured on an Agilent 6530 TOF LC-MS spectrometer with a Porshell 120 EC-C18 column (4.5 × 50 mm, 2.7 μm, Agilent Technologies, Waldbronn, Germany). Column chromatography (CC) was performed using silica gel, 200–300 mesh (Qingdao Marine Chemical Company, Qingdao, China), and Sephadex LH-20 (YMC. CO., LTD, Kyoto, Japan). Precoated silica gel GF-254 plates (Qingdao Marine Chemical Company, Qingdao, China) were used for analytical TLC. NMR solvents were purchased from Sigma-Aldrich (Merck, Shanghai, China).

### 3.2. Fungal Material

The strain NF666 was isolated from seaweed collected in August 2020 from the South China Sea, Hainan Province, China. The identification of the NF666 stain was achieved by analyzing the 18S rDNA sequences that showed a great similarity to those accessible via the BLAST+2.16.0 of *Aspergillus*. The live culture of the NF666 strain was kept at the School of Medicine, Nanjing University of Chinese Medicine (China).

### 3.3. Fermentation, Extraction and Isolation

The fungus was cultured in potato dextrose broth (PDB) at 25 °C on a rotary shaker (220 rpm) for 3 days to obtain the seed culture. Large-scale fermentation proceeded in rice medium (80 g rice and 120 mL distilled water) at room temperature for 20 days after inoculation with 10 mL seed culture. The culture medium including the mycelium was extracted three times with EtOAc. After removal of the organic solvent, the crude extract (20 g) was subjected to silica gel CC using gradient elution with a mixture of CH_2_Cl_2_/MeOH (100:0, 100:1, 100:2, 100:4, 100:8, 100:16, 0:100, *v*/*v*) to yield seven fractions (Fr.1–Fr.7), respectively. Fr.3 was subjected to further CC (SiO_2_; petroleum ether (PE)/AcOEt (10:1, 8:1, 5:1, 3:1, 1:1, 0:1, (*v*/*v*)) to yield subfractions Fr.5.1-5.6. Fraction 5.2 was fractionated by Sephadex LH-20 with CH_2_Cl_2_-MeOH (1:1) and further purified by semi-preparative HPLC to yield **1** (76% MeOH-H_2_O, 2.5 mL/min, 5.5mg, t_R_ = 18.0 min) and **2** (76% MeOH-H_2_O, 2.5 mL/min, 3.0 mg, t_R_ = 22.0 min).

### 3.4. Antibacterial Assay

Minimum inhibitory concentrations (MIC) were determined for the antibacterial activity against a series of pathogens including *Pseudomonas aeruginosa*, *Escherichia coli*, *Staphylococcus aureus*, *Bacillus subtilis*, and *Pectobacterium carotovorum* subsp. *Carotovorum*. The compounds were prepared in a 10 mM solution and successively diluted in a gradient to obtain 5 mM, 2.5 mM, and 1.25 mM solutions. All tested bacteria were activated and cultured in LB broth at 37 °C for 12 h. The assays were performed in 96-well plates, wherein a 198 µL suspension (log phase) of bacteria was supplied with 2 µL of diluted compounds. DMSO and apramycin were used as the negative and positive controls, respectively. The MIC was defined as the lowest concentration of a compound that inhibited visible bacterial growth [25].

## 4. Conclusions

Two new decalin-tetramic acid hybrid metabolites, zopfiellamides C (**1**) and D (**2**) were isolated from the marine-derived fungus *Aspergillus* sp. NF666. Their structures were unambiguously determined by an extensive analysis of HRESIMS and NMR data. Compounds **1** and **2** exhibited potent antibacterial activity against four pathogens, with MIC values of about 12.5 μΜ. Moreover, we proposed a plausible biosynthetic pathway for zopfiellamide D (**2**).

## Figures and Tables

**Table 1:** The ^1^H NMR (400 MHz) and ^13^C NMR (100 MHz) data of **1** and **2** in acetone-*d*_6_ (*J* in Hz).

Position	1		2	
	*δ*_C_, Type	*δ*_H_, Multi. (*J* in Hz)	δ_C_, Type	*δ*_H_, Multi. (*J* in Hz)
1	174.8, C	-	172.9, C	-
2	115.7, CH	7.11 (d, 16.1)	115.8, CH	7.10 (d, 16.3)
3	164.9, CH	7.62 (d, 16.1)	163.1, CH	7.52 (d, 16.3)
4	43.6, C	-	43.4, C	-
5	50.9, CH	1.56 (q, 6.8)	50.8, CH	1.55 (q, 6.8)
6	136.5, C	-	136.5, C	-
7	126.0, CH	5.13 (s)	125.9, CH	5.13 (s)
8	41.4, CH	1.83 (m)	41.4, CH	1.82 (m)
9	34.8, CH_2_	1.17 (m), 1.79 (m)	34.9, CH_2_	1.18(m), 1.78 (m)
10	27.6, CH_2_	1.73 (m)	27.6, CH_2_	1.73 (m)
11	39.0, CH_2_	1.22 (m), 1.68 (m)	39.0, CH_2_	1.22 (m), 1.68 (m)
12	36.5, CH	1.44 (m)	36.5, CH	1.43 (m)
13	49.3, CH	1.44 (m)	49.3, CH	1.41 (m)
14	17.9, CH_3_	1.18 (s)	17.9, CH_3_	1.17 (s)
15	16.8, CH_3_	1.01, d (7.0)	16.8, CH_3_	1.01, d (7.0)
16	22.3, CH_3_	1.67 (s)	22.3, CH_3_	1.67 (s)
17	23.1, CH_3_	0.82 (d, 5.7)	23.1, CH_3_	0.82 (d, 5.8)
2′	174.2, C	-	175.0, C	-
3′	100.1, C	-	100.7, C	-
4′	197.4, C	-	195.5, C	-
5′	65.0, CH	3.93 (d, 6.7)	65.1, CH	3.86 (dd, 6.0, 3.0)
6′	39.2, CH_2_	2.02 (m) 2.39 (dd, 2.3, 14.7)	39.5, CH_2_	2.25 (dd, 2.9,15.0) 2.48 (dd, 6.0,15.0)
7′	74.0, C	-	73.2, C	-
8′	177.3, C	-	177.3, C	-
9′	27.2, CH_3_	1.47 (s)	28.0, CH_3_	1.45 (s)
10′	27.1, CH_3_	2.98 (s)	27.8, CH_3_	2.97 (s)

**Table 2:** Antibacterial activities of zopfiellamides C (**1**) and D (**2**) against a panel of bacterial pathogens.

Compound	MIC (μM)	
	1	2
*Pseudomonas aeruginosa*	12.5	25.0
*Staphylococcus aureus*	12.5	12.5
*Bacillus subtilis*	>100	>100
*Escherichia coli*	12.5	12.5
*Pectobacterium carotovorum* subsp. *Carotovorum*	12.5	25.0

## Data Availability

Data are contained within the article and Supplementary Materials.

## Supplementary Materials

The following supporting information can be downloaded at: https://www.mdpi.com/article/10.3390/molecules30071502/s1: Figures S1–S8: HRESIMS, ^1^H NMR, ^13^C NMR, DEPT, COSY, HSQC, HMBC, and NOESY spectra for compound **1**; Figures S9–S16: HRESIMS, ^1^H NMR, ^13^C NMR, DEPT, COSY, HSQC, HMBC, and NOESY spectra for compound **2**.
