# Supplementary material for: Zopfiellamides C and D, New Decalin-Type Tetramic Acid Derivatives from the Marine-Derived Fungus Aspergillus sp. NF666
Source: Molecules. 2025 Mar 28;30(7):1502. doi: 10.3390/molecules30071502 (PMC11990187; doi:10.3390/molecules30071502)
Supplement: Supplementary file 1 [file molecules-30-01502-s001.zip › molecules-3470754-supplementary.pdf]

List of contents:

18S rRNA sequence of strain

Fig. S1. HRESIMS spectrum of compound **1**

Fig. S2. ^{1}H NMR spectrum (400 MHz) of **1** in acetone-d_6

Fig. S3. ^{13}C NMR spectrum (100 MHz) of **1** in acetone-d_6

Fig. S4. DEPT spectrum of compound **1** in acetone-d_6

Fig. S5. ^{1}H-^{1}H COSY spectrum of **1** in acetone-d_6

Fig. S6. HSQC spectrum of **1** in acetone-d_6

Fig. S7. HMBC spectrum of **1** in acetone-d_6

Fig. S8. NOESY spectrum of **1** in acetone-d_6

Fig. S9. HRESIMS spectrum of compound **2**

Fig. S10. ^{1}H NMR spectrum (400 MHz) of **2** in acetone-d_6

Fig. S11. ^{13}C NMR spectrum (100 MHz) of **2** in acetone-d_6

Fig. S12. DEPT spectrum of compound **2** in acetone-d_6

Fig. S13. ^{1}H-^{1}H COSY spectrum of **2** in acetone-d_6

Fig. S14. HSQC spectrum of **2** in acetone-d_6

Fig. S15. HMBC spectrum of **2** in acetone-d_6

Fig. S16. NOESY spectrum of **2** in acetone-d_6

18S rRNA sequence of strain

AATGATCTTTCCGTAGGGGGGGACTGCGGAAGGATCATTACCGAGTGAGGGTCCTC GTGGCCCAACCTCCCACCCGTGACTACTGTACCACTGTTGCTTCGGCGGGCCCGCCAG CCTAGCTGGCCGCCGGGGGGCTTCTGCCCCCGGGCCCGCGCCCGCCGGAGACCCCAA CACGAACACTGTTTCTGAAAGCCTGTATGAATCCGATTCTTTGTAATCAGTTAAAACTT TCAACAATGGATCTCTTGGTTCCGGCATCGATGAAGAACGCAGCGAAATGCGATAACT AATGTGAATTGCAGAATTCAGTGAATCATCGAGTCTTTGAACGCACATTGCGCCCCCTG GTATTCCGGGGGGCATGCCTGTCCGAGCGTCATTACTGCCCTCAAGCCCGGCTTGTATT GGGTCCTCGTCCCCCCGGGGACGGGCCCGAAAGGCAGCGGCGGCACCGCGTCCGGTC CTCGAGCGTATGGGGCTTTGTCACCCGCTCTGTAGGCCCGGCCGGCGCCAGCCCACGC AGATCATCCTTTTTTTCAGGTTGACCTCGGATCAGGTAGGGATACCCGCTGAACTTAAG CATATCATAAAGCCGGAGGAAAGGGGGTACCTACG

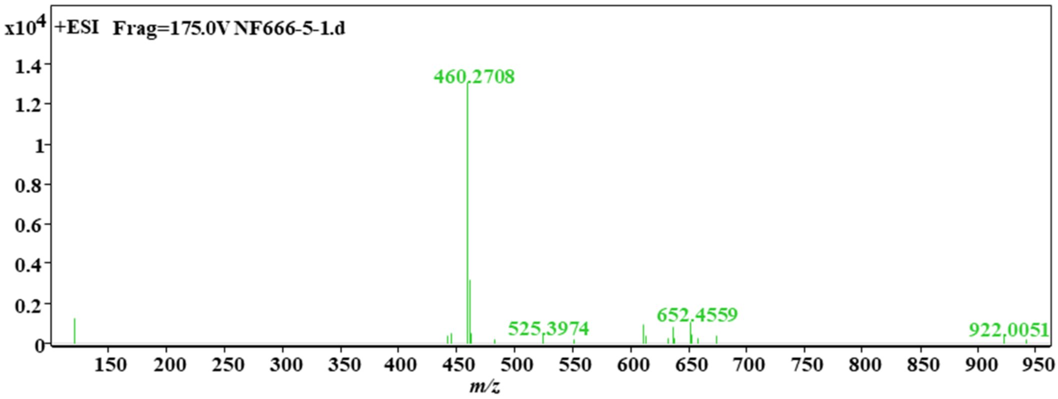

Figure S1. HRESIMS spectrum of compound **1**

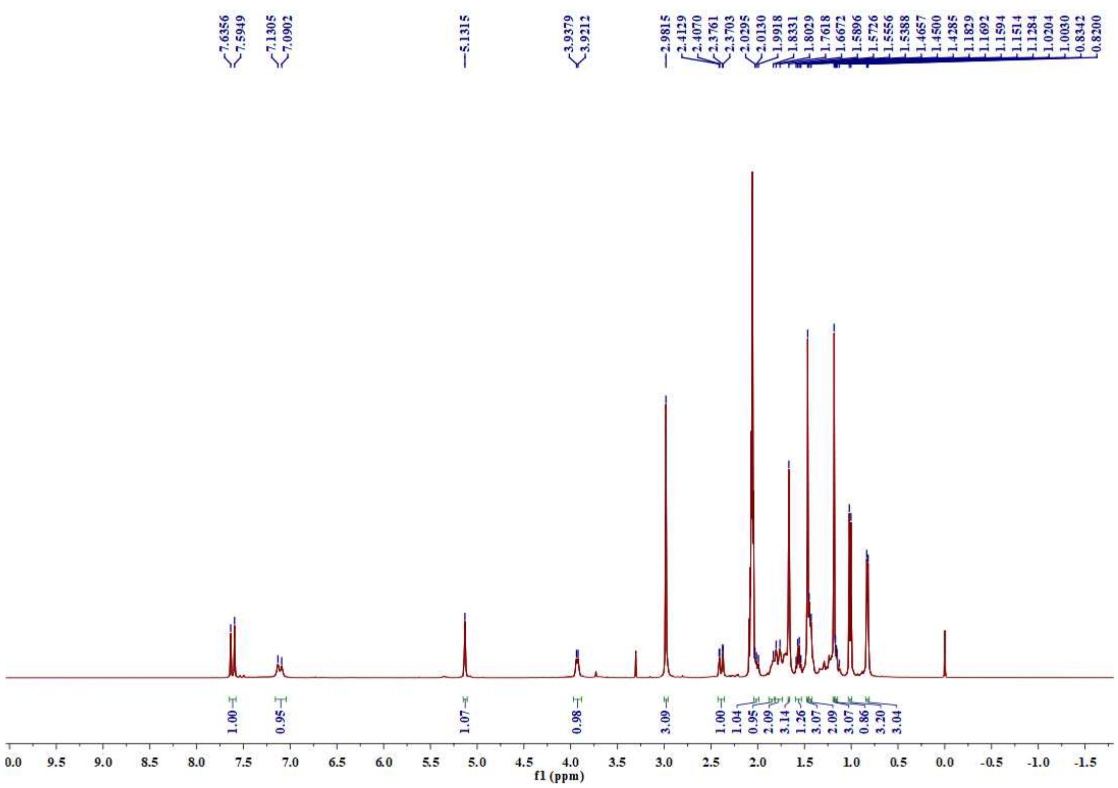

Figure S2. ^{1}H NMR spectrum (400 MHz) of **1** in acetone-d_6

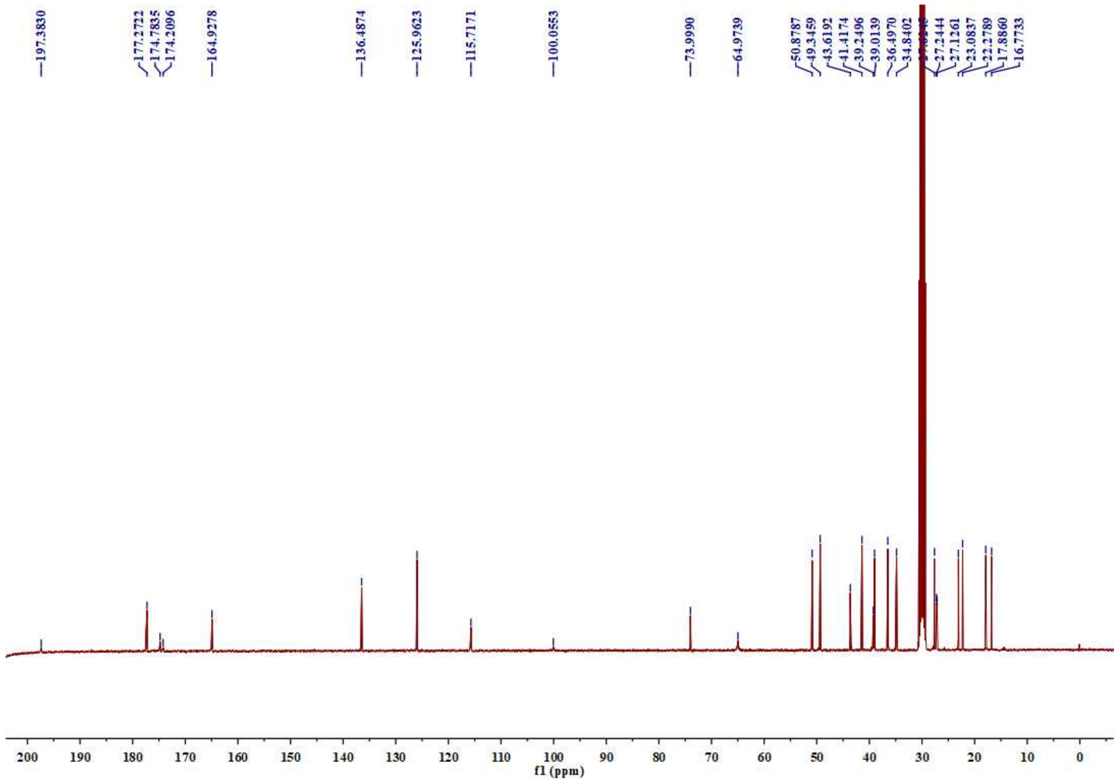

Figure S3. ^{13}C NMR spectrum (100 MHz) of **1** in acetone-d_6

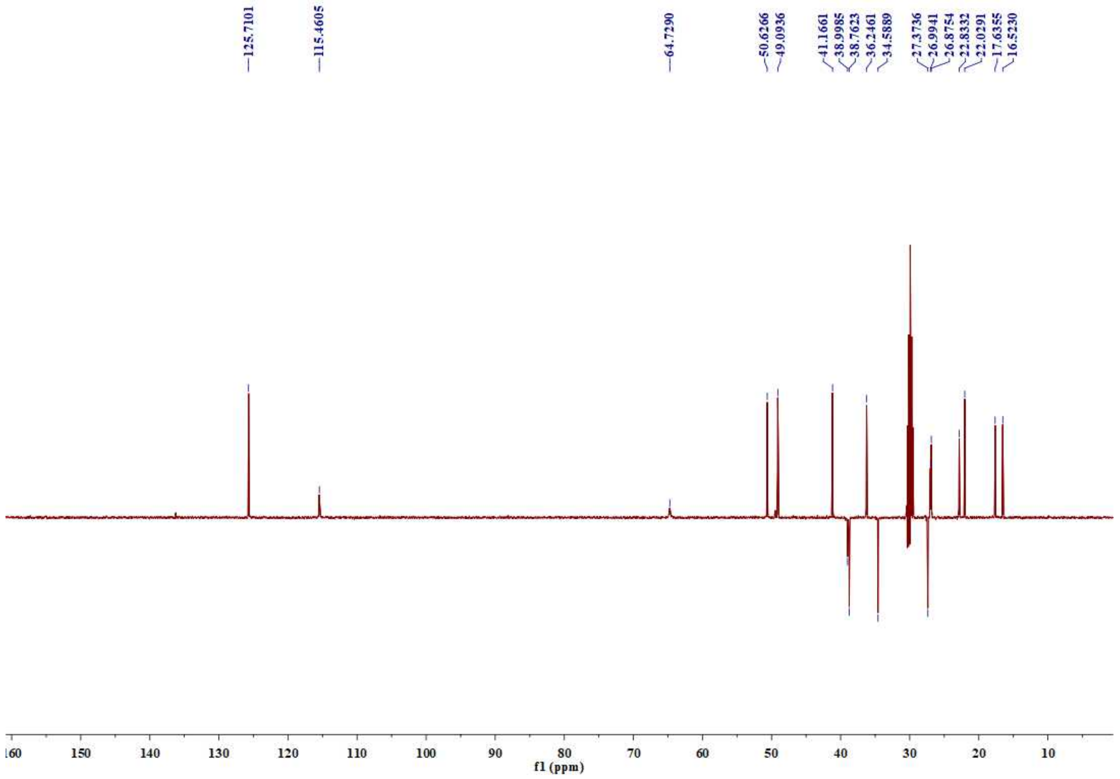

Figure S4. DEPT spectrum of compound **1** in acetone-d_6

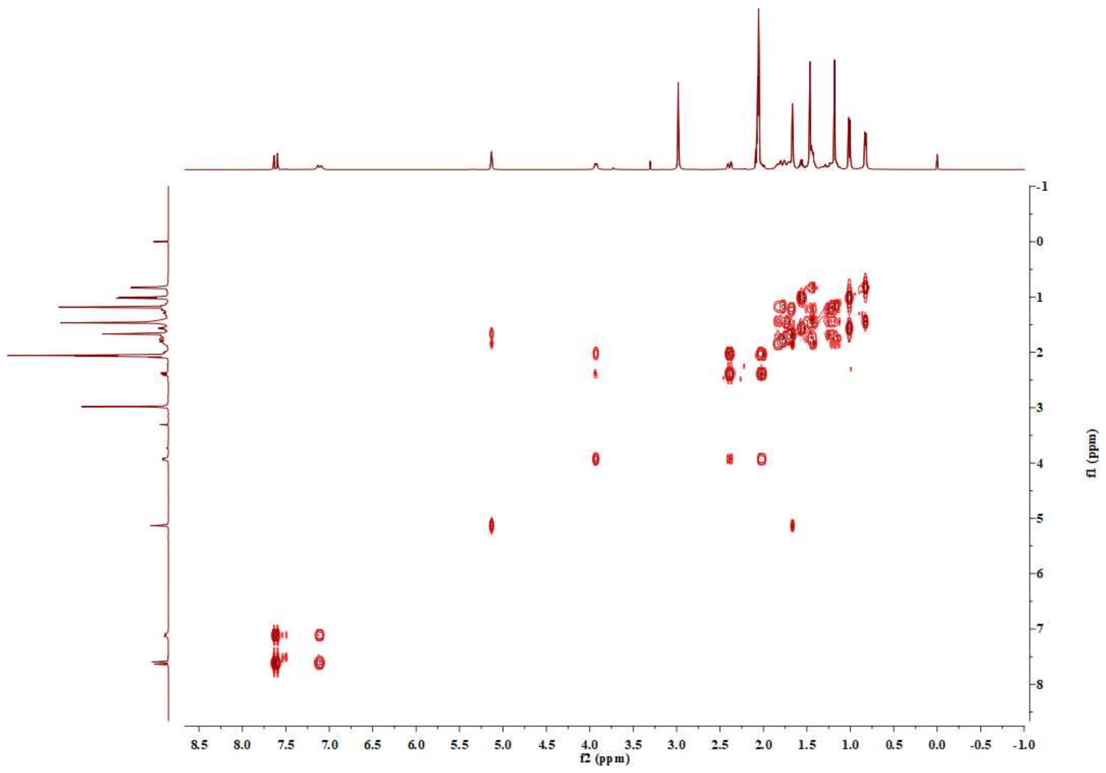

Figure S5. ^{1}H-^{1}H COSY spectrum of **1** in acetone-d_6

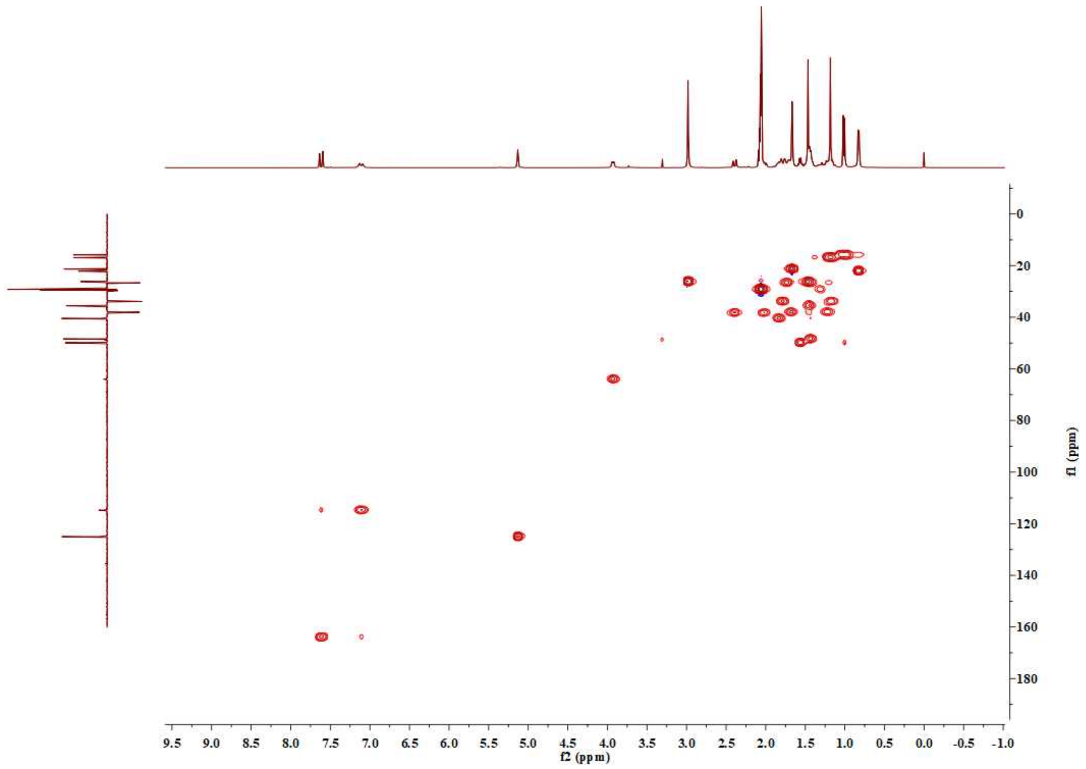

Figure S6. HSQC spectrum of **1** in acetone-d_6

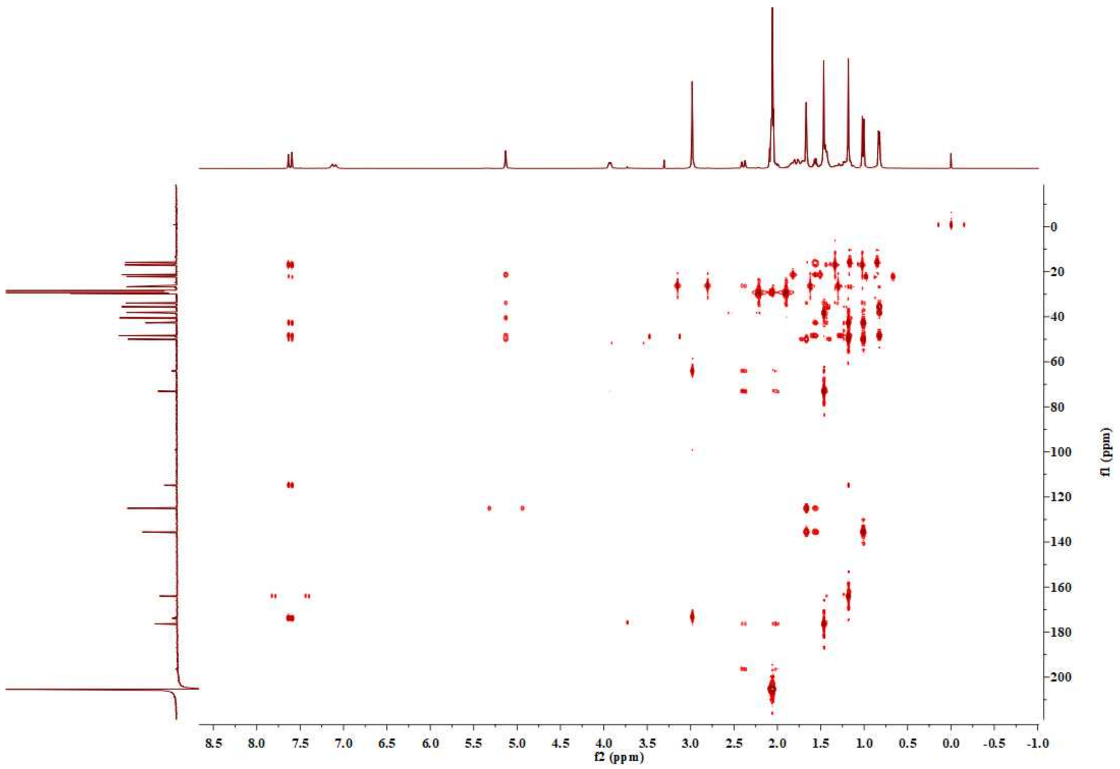

Figure S7. HMBC spectrum of **1** in acetone-d_6

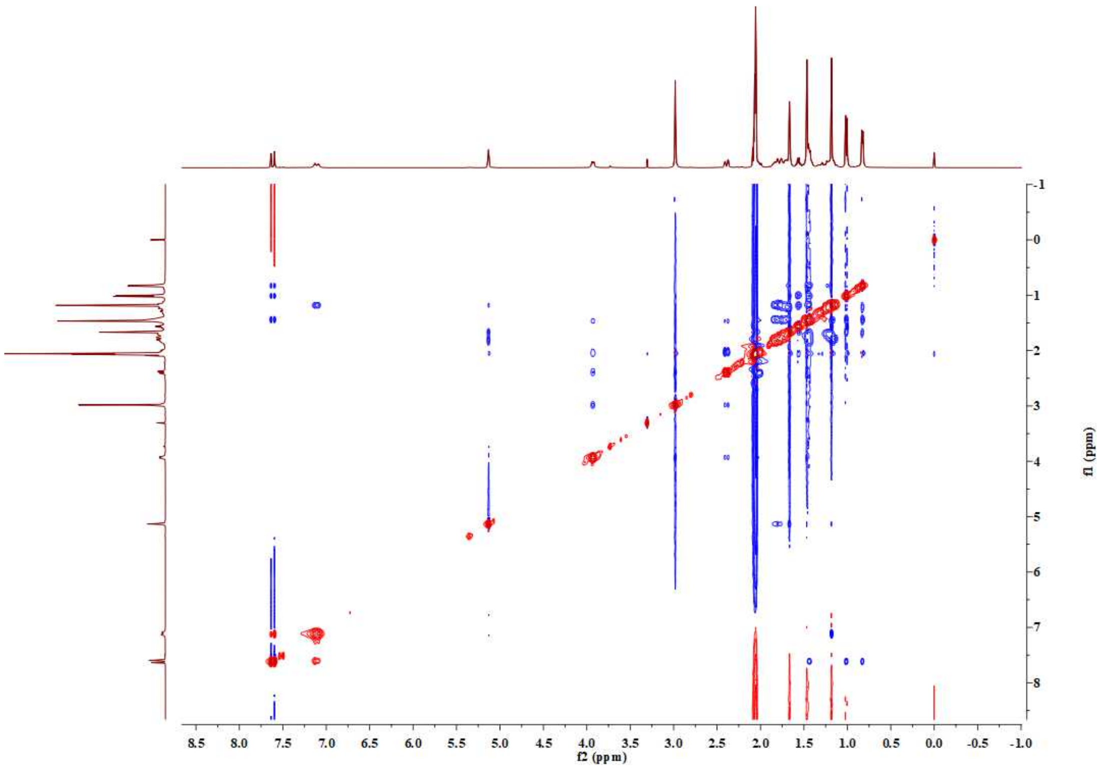

Figure S8. NOESY spectrum of **1** in acetone-d_6

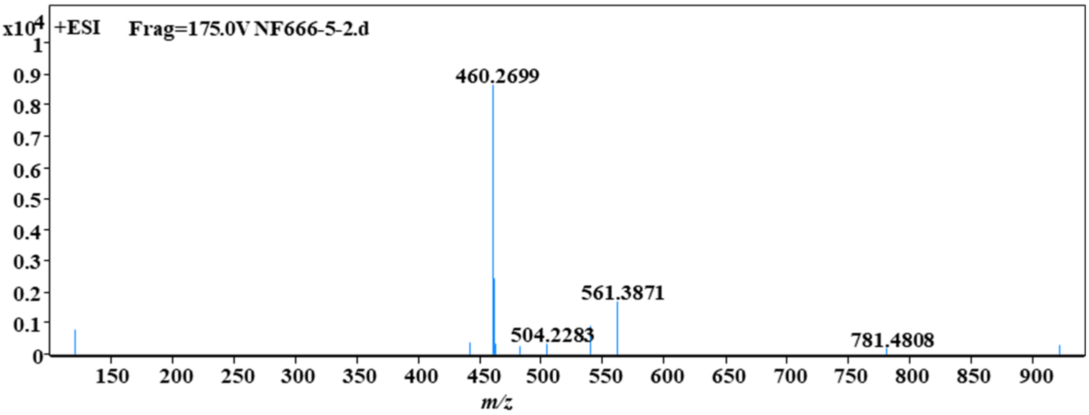

Figure S9. HRESIMS spectrum of compound **2**

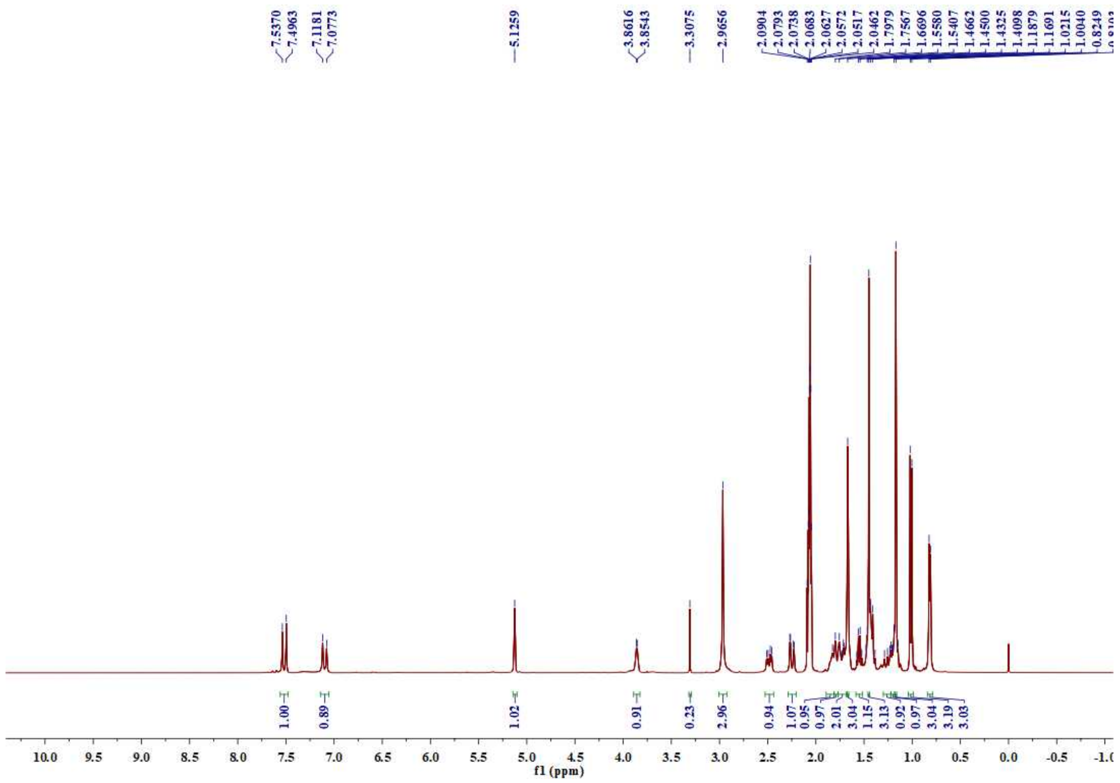

Figure S10. ^{1}H NMR spectrum (400 MHz) of **2** in acetone-d_6

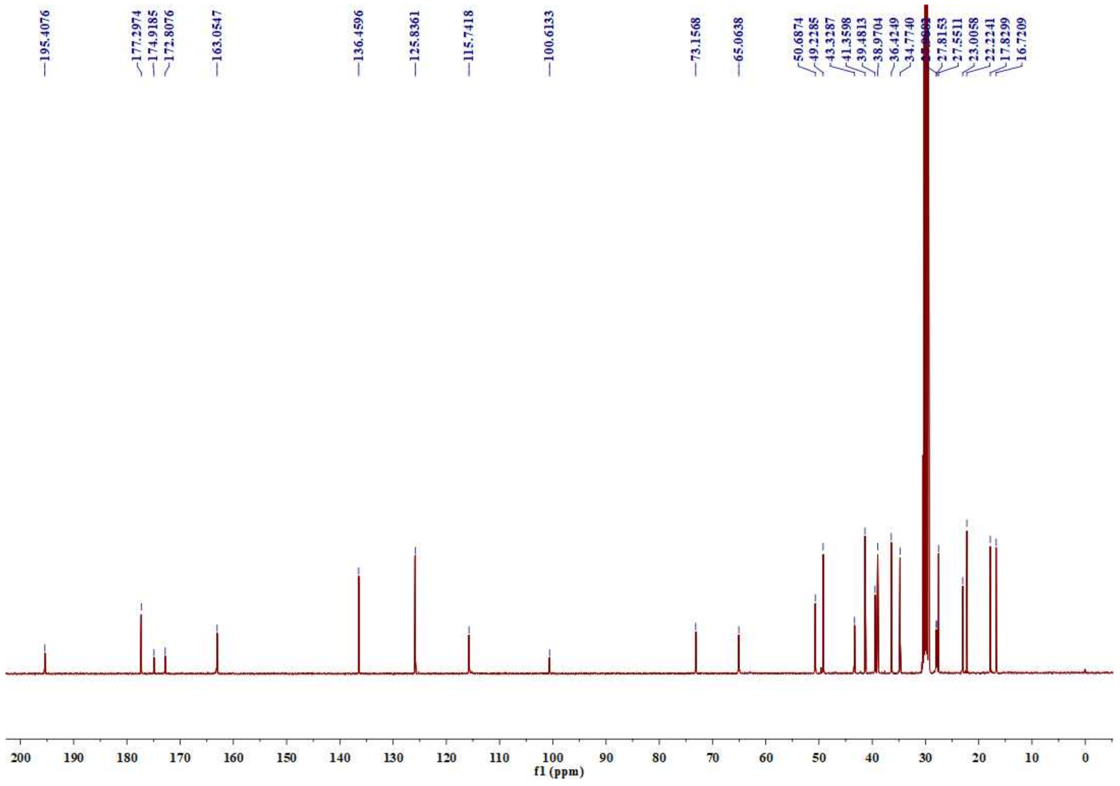

Figure S11. ^{13}C NMR spectrum (100 MHz) of **2** in acetone-d_6

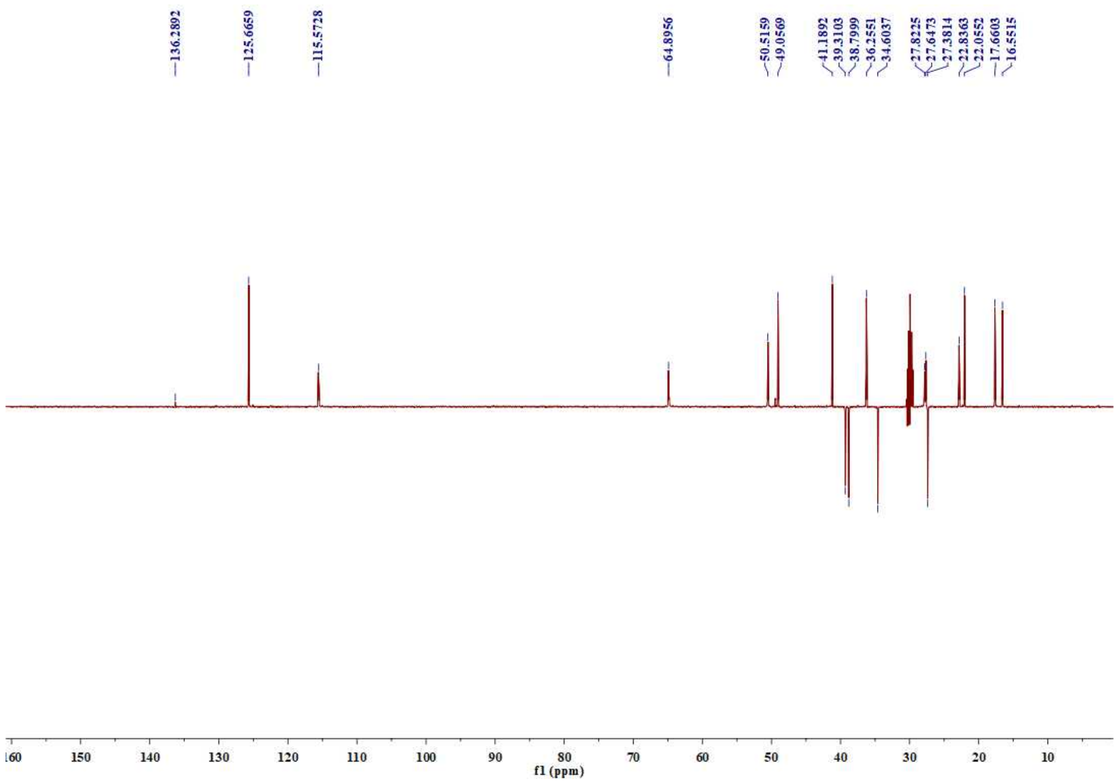

Figure S12. DEPT spectrum of compound **2** in acetone-d_6

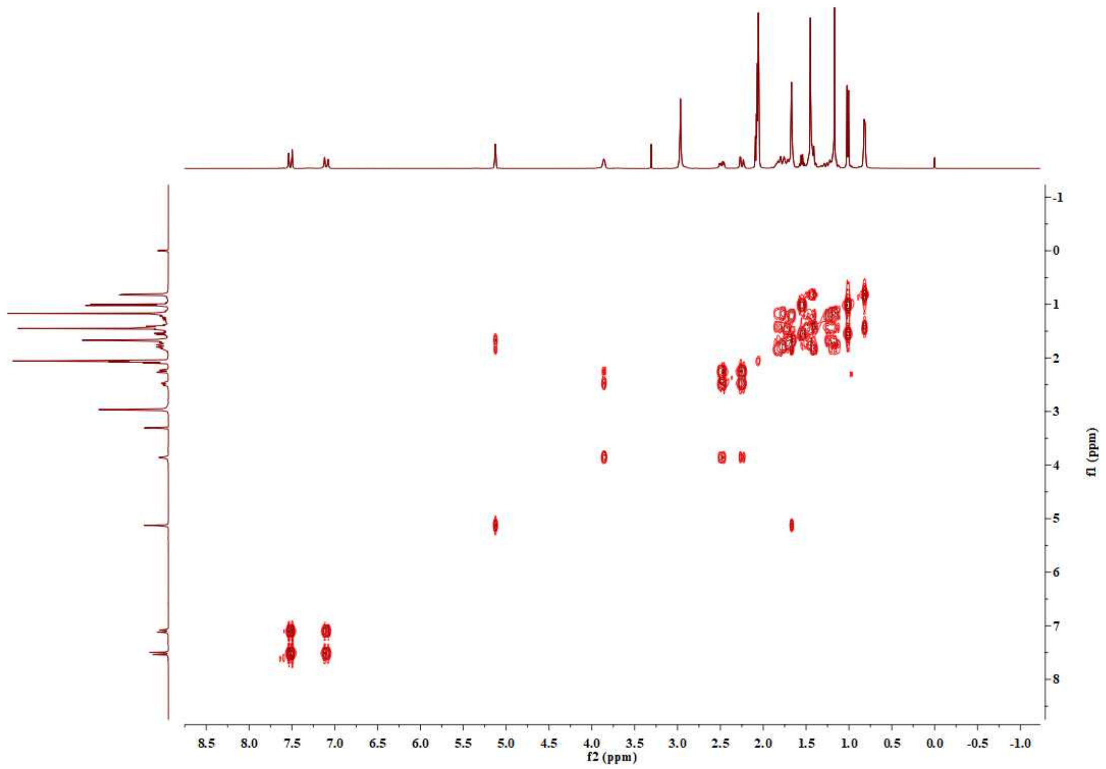

Figure S13. ^{1}H-^{1}H COSY spectrum of **2** in acetone-d_6

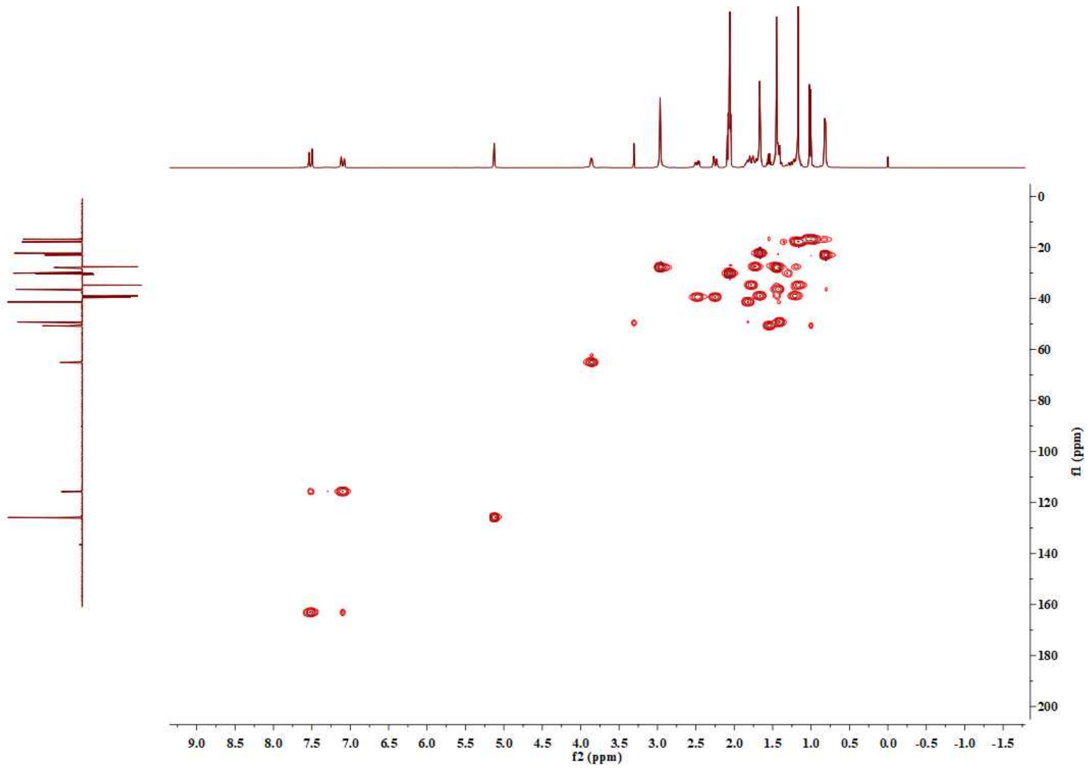

Figure S14. HSQC spectrum of **2** in acetone-d_6

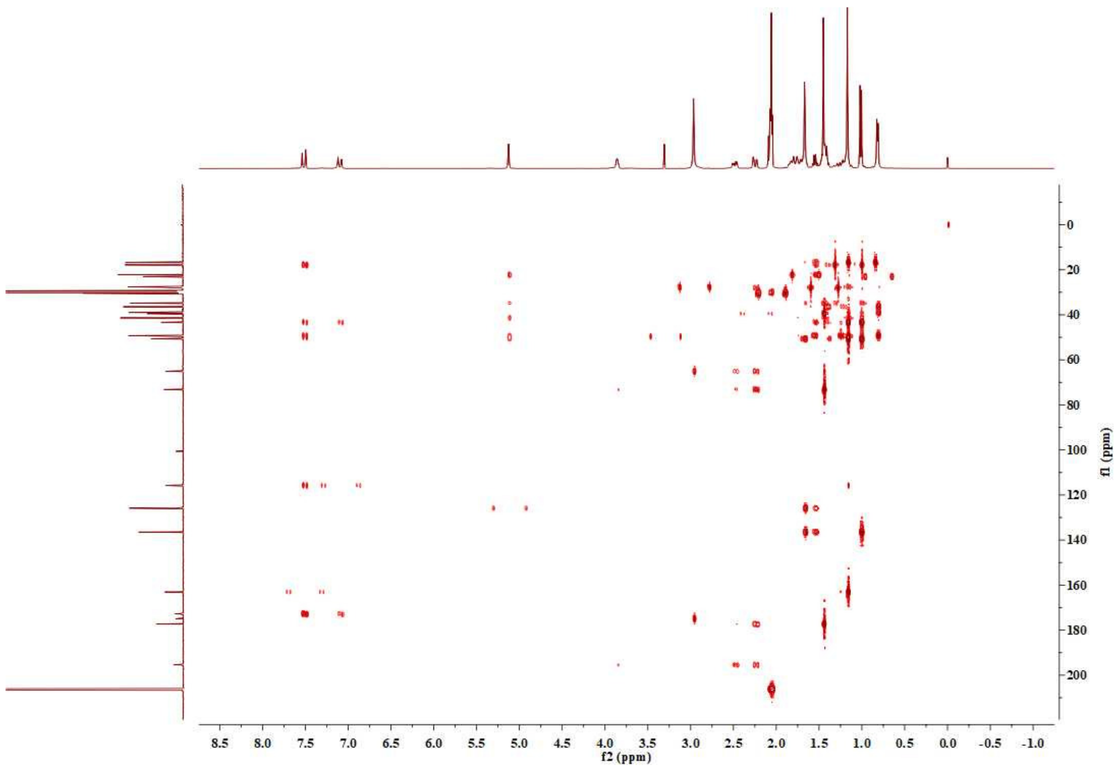

Figure S15. HMBC spectrum of **2** in acetone-d_6

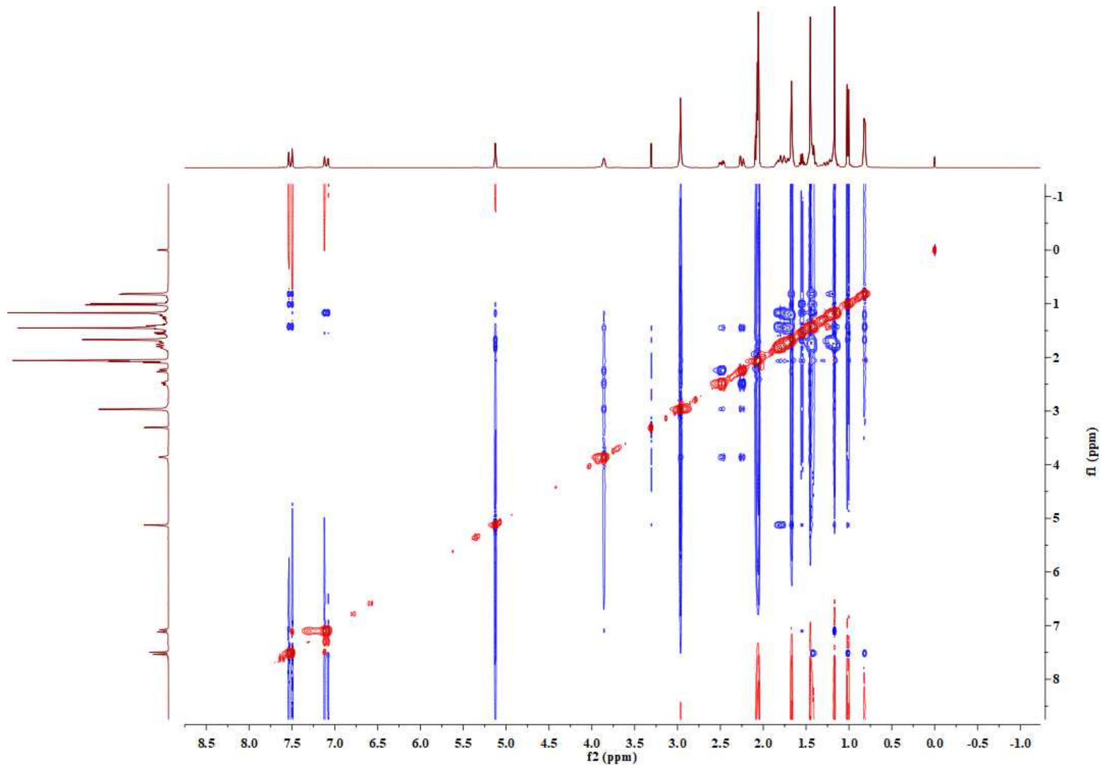

Figure S16. NOESY spectrum of **2** in acetone-d_6
